# Coordination and resource-related difficulties encountered by Quebec's public health specialists and infectious diseases/medical microbiologists in the management of A (H1N1) - a mixed-method, exploratory survey

**DOI:** 10.1186/1471-2458-12-115

**Published:** 2012-02-10

**Authors:** Charles Nhan, Réjean Laprise, Monique Douville-Fradet, Mary Ellen Macdonald, Caroline Quach

**Affiliations:** 1 Department of Pediatrics, Montreal Children's Hospital, McGill University Health Centre, C1242 - 2300 Tupper Street, Montreal, QC H3H 1P3, Canada; 2 Office of Continuing Professional Development, Fédération des médecins spécialistes du Québec, 2 Complexe Desjardins, Rm 3000, P.O. Box 216, Desjardins Station, Montreal, QC H5B 1G8, Canada; 3 Direction des risques biologiques et de la santé au travail, Institut national de santé publique du Québec, 2400 D'Estimauville Street, Room U-3141, Quebec City, QC G1E 7G9, Canada; 4 Continuing Medical Education Unit, Association des médecins spécialistes en santé publique et médecine préventive du Québec, 2 Complexe Desjardins, Rm 3000, P.O. Box 216, Desjardins Station, Montreal, QC H5B 1G8, Canada; 5 Division of Oral Health and Society, Faculty of Dentistry, McGill University, 3550 University Street, Montreal, QC H3A 2A7, Canada; 6 Continuing Medical Education Unit, Association des médecins microbiologistes et infectiologues du Québec, 2 Complexe Desjardins, Rm 3000, P.O. Box 216, Desjardins Station, Montreal, QC H5B 1G8, Canada

**Keywords:** Influenza, Pandemic, Public health emergency, Management, Physicians' perceptions, Mixed methods exploratory survey

## Abstract

**Background:**

In Quebec, the influenza A (H1N1) pandemic was managed using a top-down style that left many involved players with critical views and frustrations. We aimed to describe physicians' perceptions - infectious diseases specialists/medical microbiologists (IDMM) and public health/preventive medicine specialists (PHPMS) - in regards to issues encountered with the pandemics management at the physician level and highlight suggested improvements for future healthcare emergencies.

**Methods:**

In April 2010, Quebec IDMM and PHPMS physicians were invited to anonymously complete a web-based learning needs assessment. The survey included both open-ended and multiple-choice questions. Descriptive statistics were used to report on the frequency distribution of multiple choice responses whereas thematic content analysis was used to analyse qualitative data generated from the survey and help understand respondents' experience and perceptions with the pandemics.

**Results:**

Of the 102 respondents, 85.3% reported difficulties or frustrations in their practice during the pandemic. The thematic analysis revealed two core themes describing the problems experienced in the pandemic management: coordination and resource-related difficulties. Coordination issues included communication, clinical practice guidelines, decision-making, roles and responsibilities, epidemiological investigation, and public health expert advisory committees. Resources issues included laboratory resources, patient management, and vaccination process.

**Conclusion:**

Together, the quantitative and qualitative data suggest a need for improved coordination, a better definition of roles and responsibilities, increased use of information technologies, merged communications, and transparency in the decisional process. Increased flexibility and less contradiction in clinical practice guidelines from different sources and increased laboratory/clinical capacity were felt critical to the proper management of infectious disease emergencies.

## Background

On June 11th, 2009, the World Health Organization (WHO) declared that the circulating influenza A (H1N1) strain had reached the pandemic level; [1] Canada launched, as planned, a top-down pandemic response [2,3]. As healthcare management is a provincial responsibility, this response was carried out by provinces and territories; each assuming coordination among healthcare system stakeholders within their respective jurisdictions. Regional agencies were responsible for the implementation at the local level. Overall, the Canadian response was based on the WHO framework, which outlines essential aspects for an effective response, and included components of surveillance, healthcare response, public health intervention, communication, and command. Communication, amongst all aspects of an effective public health response [4-6], was identified as a key element: to share evidence, to aid in risk assessment, healthcare planning, and public health responses but also to encourage changes in behaviors and to convey messages [7,8].

In the province of Quebec, the vast majority of practicing physicians are members of their respective disciplinary association [9]. The Quebec's College of Physicians is legally responsible to ensure physicians' competence [10] and requires that these associations provide their members with continuing medical education (CME) focused on societal needs and in agreement with Canadian accreditation standards [11]. In line with this mandate and in the face of public criticisms [12] and members' frustrations with the overall pH1N1 management, Quebec associations of infectious diseases and medical microbiologists (AMMIQ) and of public health and preventive medicine (AMSSCQ) surveyed their members in preparation for a joint, interdisciplinary CME activity, to identify learning needs as to WHO guidelines for effective healthcare emergencies response and perceived implementation issues as experienced during the influenza A (H1N1) pandemic (pH1N1). There is scant literature about physicians' perceptions about healthcare system's management of pH1N1 [13,14]. We report the results of a secondary analysis of this exploratory survey, describing AMMIQ and AMSSCQ members' perceptions of critical issues in regards to pandemic management, and highlight suggested improvements.

## Methods

### Study population

Infectious diseases/medical microbiologists (IDMM) and public health/preventive medicine specialists (PHPMS) who were active members of either AMMIQ or AMSSCQ were eligible to participate.

### Survey tool

Two authors (CQ and MDF), involved in the management and implementation of the pandemic response at the provincial, regional, and local levels, drafted a web-based questionnaire to document physicians' perceptions on the pH1N1 response. Items on the questionnaire reflected the authors' respective medical-specialty expert knowledge of the guidelines as well as literature on effective healthcare emergencies interventions. This drafted questionnaire was then reviewed with members of each association's CME Committee (2 for AMMIQ and 3 for AMSSCQ).

The final questionnaire was composed of 4 broad questions, each with sub-questions. The first question asked whether participants were involved in the pH1N1 management (Y/N). The second question used a set of multiple-choice drop-down menus and asked participants to describe their practice profile: specialty, practice field and setting, and type of health care region. Free text fields allowed respondents who chose "other" to further describe their practice profile. The third question aimed to document types of difficulties/frustrations, if any, physicians encountered in their practice during the pH1N1 episode. A checklist of 22 items grouped in 7 categories was provided and participants were asked to check off all that applied. Each category addressed a different aspect of the pH1N1 management: at the clinical and public health level, overall crisis management, communication process, vaccination, overall management of the two pandemic waves, and issues not covered in previous categories. Within each category, physicians had the opportunity to report on issues not previously listed. At the end of each category, they were also invited to describe, in free text, issues experienced. The final question was open-ended and asked participants to suggest improvements for the management of future pH1N1-like healthcare emergencies. There were no mandatory questions.

### Data collection

The survey URL link was e-mailed on April 12th and 15th, 2010 to members of both associations. E-mail reminders were sent twice to participants and the survey was closed on May 2nd, 2010. Participation was voluntary and both respondents and non-respondents, remained anonymous.

### Analysis

Data (quantitative, qualitative) were analysed to describe and understand critical issues and suggest improvements associated with the pandemic response implementation as perceived by respondents. Descriptive statistics were used to summarize responses to multiple-choice questions. Thematic content analysis was used for qualitative data [15]. For this qualitative analysis, open coding was used to break down free text into small units (meaning units) that conveyed distinct messages, maintaining participant's original wording. Codes were assigned to these meaning units. The data was then reorganized using these codes as the unit of analysis. The codes were organized into thematic categories. These categories, created by one investigator (CN), were then reviewed and reworked by two others (CQ, RL), using team consensus. The categories were then ordered into analytic trees (themes) with branches (subthemes) grouping the categories into higher and lower levels of conceptual abstraction. Finally, using concept mapping [16], flowcharts were created in an iterative team process to explore and refine the relationships between the meaning units, codes, categories, subthemes, and themes: this concept mapping also ensured an overarching understanding of the entire data set. Two core themes were ultimately determined: Coordination and Resources. Representative verbatim quotations were then chosen to illustrate each subtheme of these core themes.

### Ethics

The anonymous survey was prepared and administered jointly by both association's CME committees in fulfilment of CME accreditation standards [11]. Preliminary survey results were presented at a one-day, interdisciplinary CME meeting organized around healthcare emergencies management and involving members of both associations (also respondents to the survey); recommendations for improvements of the healthcare response at various levels were proposed. Participants suggested that both associations advocate for those changes and that the survey results be published in support of these recommendations, which was later adopted by elected representatives of both associations. We therefore conducted "a secondary analysis of a suitably anonymized dataset that does not require ethics committee review" [17] for publication.

## Results

### Response rates and respondents' practice profile

Forty-two percent (134/317) of eligible physicians completed the survey. Five respondents were excluded, as they did not indicate their specialty. Practice profiles of the remaining 129 respondents, representing 39% (68/173) and 41% (61/147) of AMMIQ and AMSSCQ members, respectively, are described in Table 1.

**Table 1:** Practice profiles of 129 specialist physicians who responded to the survey sent to all members of Quebec associations of infectious diseases and medical microbiologists and of public health and preventive medicine

	IDMM		PHPMS	
	**All respondents N = 68**	**Respondents involved in the 2009 pH1N1 N = 62**	**All respondents N = 61**	**Respondents involved in the 2009 pH1N1 N = 40**

*Practice Field*				

Infectious Diseases	65 (97.0%)	59 (96.7%)	19 (31.7%)	19 (48.7%)

Other	2 (3.0%)	2 (3.3%)	41 (68.3%)	20 (51.3%)

*Practice Setting*				

Clinical Setting	49 (72.1%)	47 (75.8%)	1 (1.7%)	1 (2.6%)

Local Health Team	1 (1.5%)	1 (1.6%)	3 (5.0%)	1 (2.6%)

Regional Public Health Team	0	0	45 (75.0%)	34 (87.2%)

Provincial Public Health Team	1 (1.5%)	1 (1.6%)	9 (15.0%)	3 (7.7%)

Other	17 (25.0%)	13 (21.0%)	2 (3.3%)	0

*Type of Health Care Region*				

Academic	37 (55.2%)	33 (54.1%)	32 (61.5%)	21 (60.0%)

Intermediate	18 (26.7%)	17 (27.9%)	12 (23.1%)	8 (22.9%)

Peripheral	8 (11.9%)	8 (13.1%)	7 (13.5%)	5 (14.3%)

Remote	4 (6.0%)	3 (4.9%)	1 (1.9%)	1 (2.9%)

IDMM: infectious diseases specialist/medical microbiologists; PHPMS: public health and preventive medicine specialists

A greater proportion of IDMM (91.2%) than PHPMS (65.6%) were involved in pH1N1 management. Practice profiles of IDMM involved and not involved in pH1N1 management were similar. PHPMS involved in pH1N1 management were more likely to practice in the field of infectious disease (48.7% vs. 31.7%) and in a regional public health team (87.2% vs. 75.0%) than those who were not. Other practice characteristics were similar for both subgroups.

### Frequency of issues experienced during the pH1N1

Table 2 summarizes responses to the checklist of potential issues for the 102 respondents involved in the pH1N1 episode. Overall, 85.3% (n = 87) of respondents encountered difficulties or experienced frustrations in their practice during pH1N1 and this proportion was similar for both specialties. Issues related mainly - for IDMM - to laboratories and infection prevention and control, vaccine availability, communication process (clinical practice guidelines' [CPG] dissemination, and communication routes), and with the overall management of the two pandemic waves. PHPMS reported problems mainly with the decision-making process in the prioritization and vaccination of high-risk groups. In addition, more than 50% reported issues with the top-down management process, communication processes (CPGs' dissemination and communication routes), and patient management at the public health level (expert committees, case reporting, and epidemiological investigation).

**Table 2:** Difficulties/frustrations reported by 102 specialist physicians who were involved in the management of Quebec's 2009 pH1N1

Have you experienced difficulties/frustrations?	**IDMM (n = 62) **n (%)	**PHPMS (n = 40) **n (%)
***Overall categories***	***52 (84.0%)***	***35 (87.5%)***

Patients' management - clinical level		

*Overall*	*49 (79.0%)*	*10 (25%)*

Beds management	16 (25.8%)	0

Case reporting	19 (30.6%)	2 (5.0%)

Laboratories	40 (64.5%)	3 (7.5%)

Patients care	25 (40.3%)	1 (2.5%)

Treatment	24 (38.7%)	2 (5.0%)

Infection prevention and control	36 (58.1%)	6 (15.0%)

Other	1 (1.6%)	0

Patients' management - Public Health level		

*Overall*	*28 (45.2%)*	*22 (55%)*

Expert committees	12 (19.4%)	9 (22.5%)

Case reporting	12 (19.4%)	7 (17.5%)

Epidemiological investigations	9 (14.5%)	10 (25.0%)

Neuraminidase inhibitors prescriptions	17 (27.4%)	5 (12.5%)

Other	2 (3.2%)	8 (20.0%)

Crisis management		

*Overall*	*28 (45.2%)*	*24 (60%)*

Top-down	23 (37.1%)	22 (55.0%)

Quebec-Canada	11 (17.7%)	4 (10.0%)

Other	1 (1.6%)	2 (5.0%)

Communication process		

*Overall*	*34 (54.8%)*	*24 (60%)*

Clinical practice guidelines (content)	19 (30.6%)	11 (27.5%)

Dissemination of clinical practice guidelines	26 (41.9%)	13 (32.5%)

Communication routes	25 (40.3%)	12 (30.0%)

Other	2 (3.2%)	2 (5.0%)

Vaccination		

*Overall*	*35 (56.4%)*	*25 (62.5%)*

Availability of vaccine	21 (33.9%)	10 (25.0%)

Groups at risk	18 (29.0%)	17 (42.5%)

Number of dosage	11 (17.7%)	3 (7.5%)

Administration approach	16 (25.8%)	8 (20.0%)

Other	5 (8.1%)	4 (10.0%)

Management of pandemic waves		

*Overall*	*31 (50%)*	*11 (27.5%)*

Availability of epidemiological data on cases	11 (17.7%)	7 (17.5%)

Cases management	9 (14.5%)	3 (7.5%)

Laboratories organization	27 (43.5%)	1 (5.0%)

Other	1 (1.6%)	3 (7.5%)

Other issues not previously covered		

*Overall*	*7 (11.2%)*	*6 (15.0%)*

IDMM: infectious diseases specialist/medical microbiologists; PHPMS: public health and preventive medicine specialists

### Qualitative Analysis

**Figure 1:**
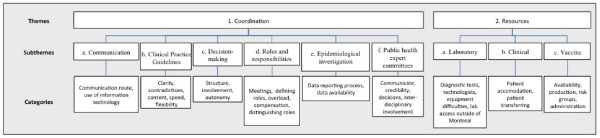
Classification of issues and suggestions mentioned by 62 specialist physicians* who were involved in the management of Quebec's 2009 A (H1N1) pandemic.* Infectious diseases specialist/medical microbiologists and public health and preventive medicine specialists.

Sixty-two of 102 (37 IDMM and 25 PHPMS) respondents (60.7%) involved in pH1N1 provided written comments. Breaking down these comments resulted in 244 distinct meaning units. Figure 1 illustrates the hierarchy of groupings that was developed from coding these meaning units into categories, subthemes, and themes. Overall, comments could be grouped under two core themes: coordination, at all levels of implementation of the pandemic response, and availability of resources required to manage the pandemic. Open codes associated with coordination (n = 180) were more frequent than those relating to resources (n = 64). The following sections report the results for each of the two core themes and their subthemes. Representative verbatim quotations of the subthemes are provided in Table 3.

**Table 3:** Representative verbatim quotations (please note: these are examples; the content of some quotations covers more than one category and thus may have appeared in multiple subthemes in our analysis)

Themes and Subthemes	Comments
Coordination	

a) Communication	"There was too much information, and too many sources" "There needs to be better coordination between the two specialties" "Difficult to communicate changing recommendations to different services" "Use the current network rather than creating a parallel network"

b) Clinical Practice Guidelines	"The waiting times to get CPG was long, and like already mentioned, documents were coming from everywhere" "Changing and contradicting CPG complicated the situation when it came time to disseminate to other health professionals and sometimes rendered the infection control guidelines less credible since they were constantly changing" "CPGs were not adjusted based on clinical reality"

c) Decision-Making	"Very little flexibility... Again, the decisions were unclear and not very well explained" "Lack of latitude at the regional level" "Too many stakeholders and too many messages" "Give more autonomy to the regional-local levels due to differences between different areas" "It is clear that an interdisciplinary expert committee should work together on the management of pandemics and other infectious disease emergencies"

d) Roles and Responsibilities	"It is frustrating to not be remunerated for the overwhelming number of calls answered and for infection control management" "There needs to be a better distinction between hierarchical roles and expert roles" "Avoid having too many meetings and instead have a better, more transparent structure that avoids daily (and multiple) changes" "Clarify roles and responsibilities quickly at the start of crises to the different parties involved"

e) Epidemiologic Investigation	"There was a big problem in accessing data (local cases: clinical presentation, severity, etc.)" "Lack of information in the beginning, late access to pertinent Quebec epidemiological data"

f) Public Health Expert Advisory Committees	"More openness from experts and less closure of government leaders, professional associations, etc." "Very little information given about the experts on advisory committees and from the different levels of government and public health" "Difficulty with decisions and conclusions of the committees and treatment recommendations"

	"Delay in the transmission of clinical practice guidelines from the Committee of...*"

Clinical Resources	

a) Laboratory Resources	"Long delays in obtaining results" "Regional labs should have access to proper diagnostic technologies" "The number of lab technologists available is insufficient" "Allow diagnostic PCR analysis at the local level, which will allow faster results and thus better management of patient beds"

b) Patient Management	"Lack of individual rooms" "Difficulties encountered in transferring patients to intensive care" "Patients were referred directly to our hospital's emergency department without prior evaluation"

c) Vaccination Process	"Peculiar recommendations for different risk groups" "Too late to have the most impact" "Late vaccination of the general population" "Late access to vaccines; only supplied by one company; lack of non-adjuvanted vaccine for target groups"

* We did not want to name the actual committee

### Coordination

Issues and suggested improvements with coordination comprised the following subthemes:

a Communication: A slow communication process, an overwhelming number of communication sources, and an overwhelming number of divergent messages, sometimes lacking clarity, were identified as the main problems. Respondents suggested that these issues were in part due to communication routes used to relay information. Participants mainly suggested improvements to communication management such as greater centralization and use of the Internet instead of teleconferences.

b Clinical Practice Guidelines (CPG): Respondents found that CPGs' content was inconsistent between the different pandemic management levels and advisory committees; physicians were confused as to which to follow, especially when contradictory. The changing nature and the slow dissemination of these CPGs were also perceived as problematic. CPGs were perceived as too rigid to accommodate particular regional and local situations.

c Decision-Making: Physicians expressed unhappiness with the top-down management model and speed of decision-making, which was associated by some to the large number of people involved at the top administrative level. Physicians also found that there was a lack of autonomy and transparency in the decision-making process. Suggestions were made to involve more medical specialists in the decision-making process and to increase autonomy at the regional and local levels.

d Roles and Responsibilities: Physicians complained about increased workload related to pandemic activities, such as meetings attendance that they found inefficient. Some found financial compensations inadequate for the additional workload. Respondents also mentioned that the exact role of the different actors involved in the pandemic was unclear, which generated confusion in the local management of the pandemic. Suggestions were to improve meetings' structure and to better define roles at the beginning of a healthcare emergency.

e Epidemiologic Investigation: Public health epidemiologic investigations were mentioned as an issue, in particular the changing nature of the case report form. There was also a concern with how surveillance, modelling and analysis of data were handled and with lack of timely data feedback to the local level. The main suggestion was to increase processes transparency and improve local access to data.

f Public Health Expert Advisory Committees: Respondents were mainly concerned with the lack of communication between physicians in the field and expert advisory committees. They also questioned the credentials of committees' members and their decisions. Needs of specific regions were felt as neglected. Suggestions included increasing speed of dissemination of advisory committees decisions and provision of committee members credentials, as well as increased involvement of physicians from various disciplines in committees' decision-making process.

### Resources

This core theme included laboratory-related resources, patient management, and vaccination process.

a Laboratory Resources: Limited availability of diagnostic material and human resources and poor access to diagnostic tests such as nucleic acid amplification tests (e.g. PCR) were raised as issues.

b Patient Management: Many patients with influenza-like symptoms were sent to emergency rooms without prior evaluation, resulting in overburdened emergency rooms. Respondents suggested that those patients be evaluated elsewhere. Some also mentioned a lack of hospital single rooms to accommodate patients with pH1N1, as well as difficulty in transferring patients.

c Vaccination Process: Vaccines arrived late after the onset of the second wave of the pandemic and notifications of availability were last minute. Physicians expressed disagreement with high-risk group prioritization, especially in regards to school-aged children and the elderly population, who were targeted late in the vaccination campaign. Respondents proposed approaching group prioritization based on a better risk assessment. Other suggestions included earlier accessibility to the vaccine for the general public and the need for specialized clinics to serve chronically ill patients. Physicians would also have liked to receive more information on the vaccine.

## Discussion

In this study, the majority of physicians who answered this exploratory survey reported difficulties or frustrations in their practice during the pH1N1. Quantitative results suggest that some of the reported issues, such as access to laboratory material, were specialty-specific while others, such as communication processes, were experienced by both groups of physicians.

Exploration of the qualitative data contributed greatly to the interpretation of the quantitative data. The qualitative analysis suggested that most difficulties experienced during pH1N1 were related to coordination of response between stakeholders. Most problems were experienced within the areas of CPG, communications processes, and decision-making. Communication is especially crucial in risk management [8] but also in information transfer, such as infection control measures [18] and new data on disease processes [19]. Difficulties, resulting from too many different and contradicting sources, as well as lack of flexibility, were the main areas identified as problematic. Various advisory committees may have interpreted available evidences differently, leading to contradictions. However, trust in guidelines is highly dependent on believing that the sources of those guidelines are credible. Transparency in the decision-making process and decision makers' credentials is crucial.

As in other studies [20-22], efficient communication between various actors was felt to be important. It was suggested that the excessive amount of communication sources and messages might be solved by streamlining communications and through better use of the Internet rather than traditional communication routes such as teleconferences. A need for increased use of newer communication technologies, facilitating transfer of information between those on the front lines and authorities, had been advocated for effective use of CME during outbreaks and to develop flexible plans [22].

In a public health emergency, actors involved must acknowledge their roles and responsibilities. Physicians, who are usually autonomous professionals with important decision-making freedom, seemed to have difficulty with the top-down managerial style that was imposed with the implementation of the pandemic response. There may have been a lack of communication about the managerial approach that would be implemented. However, the Roundtable on Healthcare and Emergency Service Sector Pandemic Preparedness, reported that top-down is essential in emergencies management, but that a bottom-up method of feedback is also needed to allow adaptation to varying circumstances [5]. Flexibility in guidelines and in the decision-making process is also necessary to enable adaptation by allowing faster changes [20].

As previously reported [22,23], participants suggested that a mixed group of experts including top academic experts [23] in collaboration with front lines of care, and the public health sector would be beneficial [24]. Public health components are needed to support the command system in place to ensure evidence-based decisions and proper coordination of interventions [7]. Some authors have emphasized informational transparency in several decision-making aspects [8,14] to improve collaboration [8].

This study is based on a survey designed to develop a CME intervention and thus has limitations: we relied on a convenience sample; the questionnaire did not have established construct validity and recall bias in participants' answers is possible.

## Conclusions

This paper is one of the few reporting on physicians' perceptions on management of public health emergencies. Important highlighted areas were coordination between all involved, decision-making transparency, greater collaboration of health professionals in decision-making, greater flexibility, and a better definition of roles and credentials. Results emphasize the need to improve transparency and build stronger working relationships between physicians and health authorities. In times of emergency, a greater involvement of professional associations both in the planning of services and as a communication channel should be considered. Studies based on other qualitative research approaches (e.g., grounded theory) are needed to further understand how healthcare systems can improve the implementation of emergency response plans and empower stakeholders involved. It would also be useful to study identified gaps between national health authorities' pandemic plans and what actually happened in response at the different levels of implementation.

## Abbreviations

AMMIQ: Association des médecins microbiologistes et infectiologues du Québec; AMSSCQ: Association des médecins spécialistes en santé communautaire du Québec; PHPMS: Public Health and Preventive medicine specialists; CPG: Clinical practice guidelines; GT: Grounded theory; IDMM: Infectious disease specialists and Medical Microbiologists; pH1N1: Pandemic H1N1 influenza A virus; WHO: World Health Organization.

## Competing interests

The authors declare that they have no competing interests.

## Authors' contributions

CQ, MDF and RL developed the survey and contributed to data collection. RL and CQ determined data analyses. CN analyzed qualitative data and RL analyzed quantitative data. CQ and RL challenged coding. CN, RL and CQ wrote the manuscript. All authors contributed to data interpretation and critically reviewed the manuscript. All authors read and approved the final manuscript.

## Authors' information

At the time of the survey, CQ (an infectious diseases specialist and medical microbiologist) and MDF (a public health specialist) were chairing their respective CME committees. RL was acting as an expert CME research consultant for AMMIQ and AMSCCQ, and CN was a pre-med student jointly supervised by CN and RL. MEM joined the research team as a qualitative methodologist.

## Pre-publication history

The pre-publication history for this paper can be accessed here:

http://www.biomedcentral.com/1471-2458/12/115/prepub
